# The return of a forgotten ally: tabletop scanning electron microscopy in the realm of bacteriology

**DOI:** 10.3389/fcimb.2025.1697696

**Published:** 2026-01-13

**Authors:** Omar Zmerli, Meriem Boukili, Sara Bellali, Jacques Bou Khalil

**Affiliations:** 1 Aix Marseille Univ, MEPHI, Marseille, France; 2 IHU Méditerranée Infection, Marseille, France; 3 Aix Marseille Univ, APHM, MEPHI, Marseille, France

**Keywords:** antimicrobial resistance, bacteriology, microbiology research, microbiota, tabletop scanning electron microscopy

## Abstract

Scanning electron microscopy (SEM) is re-emerging as an accessible method in bacteriology, driven by technological advances that produced the powerful and compact tabletop SEM. This review highlights recent advances (2015–2025) demonstrating how novel tabletop SEM delivers rapid, high-resolution, and accurate results that can transform both fundamental and clinical bacteriology. Several studies consistently demonstrate the utility of tabletop SEM in basic research, such as studying biofilms, building antibacterial coated material, and describing bacteria-environment interactions. In clinical bacteriology, diverse applications have emerged over the past few years placing the tabletop SEM at the forefront of bacterial visualization from clinical samples, reaching accurate descriptions of bacteria-antibiotic interactions and the accurate detection of bacterial morphologic changes following exposure to antimicrobial agents with dramatically reduced turnaround times. When combined with energy-dispersive X-ray spectroscopy (EDX), tabletop SEM offers insights into bacterial metabolic states and chemical composition under stress or antimicrobial treatment. In this new era of bacteriology, tabletop SEM truly marks the return of a forgotten ally, empowering the investigative arsenal with speed, robustness, and accuracy in both research and clinical practice.

## 1 Introduction

**Figure 1:**
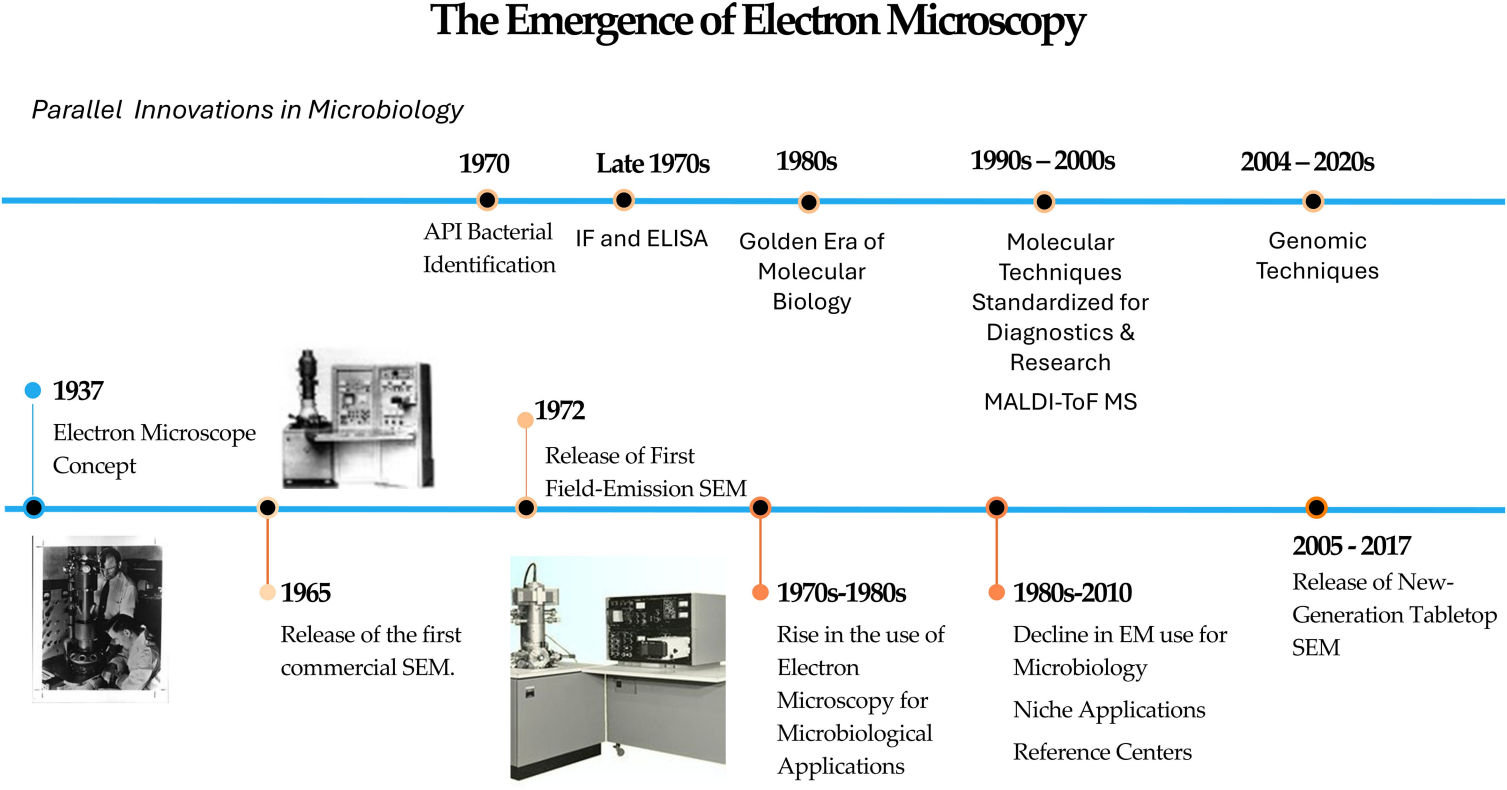
Electron microscopy developmental timeline: 1935 till 2025.

The progression of knowledge in the field of microbiology has historically been constrained by the available detection technologies, which have been in continuous development since Robert Koch’s times. In the late 19th century, Koch’s application of light microscopy enabled the precise identification of pathogens such as *Bacillus anthracis* (1876), *Mycobacterium tuberculosis* (1882) (Cambau and Drancourt, 2014), and *Vibrio cholerae* (1883) (Sravanthi et al., 2024). However, light microscopy proved insufficient for studying viral agents, which remained undetectable until the development of electron microscopy (EM) in the 1930s (Goldsmith and Miller, 2009) (**Figure 1**). EM emerged as a revolutionary technology with the first transmission electron microscope (TEM) developed by Max Knoll and Ernst Ruska in 1931-1932, fundamentally transforming the ability to visualize the microscopic world (Goldsmith and Miller, 2009). The application of electron microscopy to microbiology began almost immediately after its invention, with the first phage electron micrographs published in 1940 in Germany, proving the particulate nature of bacteriophages and revealing their complex “tadpole-like” structures comprising heads and tails (Ackermann, 2014). Throughout the 1940s and 1950s, electron microscopy flourished in microbiological research, especially after the release of the first commercial SEM in 1965. Scientists utilized various staining techniques to reveal bacterial surface topography and external structures such as flagella, while developments in sectioning and fixation methods enabled detailed examination of bacterial cell walls and internal structures (Brenner and Horne, 1959; Horne and Wildy, 1979). Therefore, EM provided unprecedented insights into bacterial morphology that were impossible to achieve with light microscopy, and which were critical for understanding bacterial pathogenicity and microbial interactions.

Despite its early success and excellent results, EM saw a decline in routine and clinical microbiological applications in the late 20th century (Goldsmith and Miller, 2009). The decline was driven by several factors, including the technical complexity of electron microscopy, which required extensive and hazardous sample preparation, and the need for high vacuum conditions that often damage biological samples (Hayat, 1981; Goldstein et al., 2017). Technological and operational challenges also contributed to this decline since most instruments were large, difficult to maintain, and required stable specialized environments. This restricted the presence of this technology to specialized facilities, making it inaccessible for most users. Additionally, traditional EM lacked sensitivity compared to newer diagnostic methods and could not analyze live specimens (Hayat, 1981).

Nevertheless, although it was established that EM was not suited for large-scale routine screening due to its time-consuming nature and the need for expertise; it remained an essential diagnostic tool for ambiguous cases, particularly during outbreaks or when dealing with emerging diseases. Unlike molecular or immunological tests that target specific pathogens, EM enables direct visualization of microbes in clinical samples without prior assumptions about their identity. This makes it invaluable for detecting new and unexpected organisms, as it can quickly help identify pathogens based on their structure. However, the technique’s effectiveness depends on proper specimen preparation, and strict adherence to biosafety protocols, especially when handling potentially dangerous agents (Hazelton and Gelderblom, 2003; Goldsmith and Miller, 2009; Jonge and de Ross, 2011).

Fortunately, technological advancements remained present in the EM field, leading to the emergence of tabletop scanning electron microscopes (SEM) in the early 21st century, marking a renaissance moment for EM in general. Most early applications were restricted to material sciences and educational activities, however, this novel technology gained traction and repositioned itself within the field of bacteriology. The development timeline of tabletop SEM technology involved multiple manufacturers pursuing parallel innovations, including Hitachi, JEOL, and ThermoFisher (Phenom). Hitachi High-Tech Corporation emerged as an early leader with their TM series, offering enhanced image quality with superior contrast and intuitive user interfaces (Hitachi technology 2006–2007, 2025). The TM4000 series featured automatic variable pressure vacuum systems and high-sensitivity detectors that enabled rapid imaging (Hitachi review, 2025; Tab letop Microscopes TM4000II/TM4000Plus II : Hitachi High-Tech Corporation, 2025). JEOL Ltd. contributed to the tabletop SEM revolution with their NeoScope™ series (Release of a New Benchtop Scanning Electron Microscope JCM-7000 Series NeoScopeTM - Benchtop SEM with seamless operation from optical to SEM imaging, and elemental analysis - | News, 2025). Thermo Fisher Scientific also released their Phenom desktop SEM series, which provided speed, ease-of-use, and great performance through a user-friendly design (Scanning electron microscopes - thermoFisher scientific, 2025). This technological breakthrough allowed tabletop SEMs to fill the critical imaging gap between light microscopy (magnifications up to 1,000×) and high-power but expensive technologies like transmission electron microscopy (magnifications of 100,000× and above). Tabletop/Desktop SEMs offered magnifications up to 20,000× with low cost and user-friendly hardware far better than many research-grade light microscopes.

**Figure 2:**
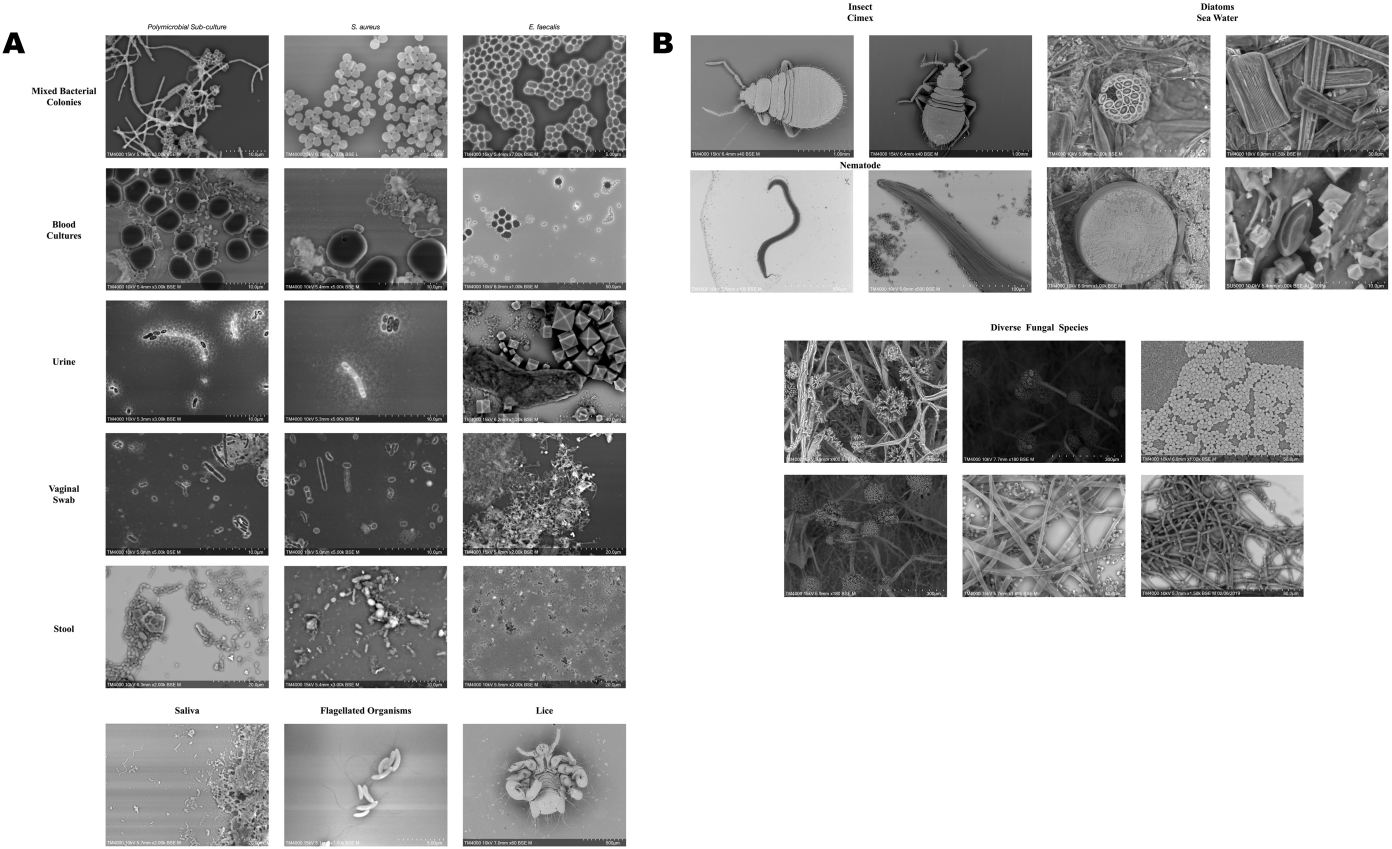
SEM images for diverse clinical **(A)** and environmental **(B)** samples observed using a tabletop scanning electron microscope. (Images courtesy of IHU Méditerranée Infection and Hitachi High-Tech).

This groundbreaking technological advancement addressed the fundamental barriers that had limited SEM accessibility by creating compact, user-friendly instruments that could operate in standard laboratory environments. This convenience factor has been crucial in expanding their adoption across biomedical applications that previously relied solely on optical microscopy. **Figure 2** showcases images acquired using a tabletop microscope for a diverse range of clinical (A) and environmental (B) samples.

This ease of use led to a surge in bacteriological research applications, from basic studies of cell wall architecture to clinical investigations of pathogen identification and drug susceptibility (Hazelton and Gelderblom, 2003; Golding et al., 2016a). Recent advances in detector technology, automation, and user-friendly interfaces mean that even non-experts can obtain publication-quality images within minutes.

Despite the major advantages brought forward by this novel instrument, it is important to acknowledge that certain technical challenges have accompanied the release of tabletop SEMs, especially when compared with conventional SEM and TEM. **Table 1** compares the main attributes of tabletop SEMs, conventional SEM, and TEM in a diagnostic context, emphasizing differences in resolution, sample preparation, and operational feasibility. These challenges, however, remain largely at the technical level and do not constitute major limitations for the intended applications. Given their balance of resolution, ease of sample preparation, and depth of information, tabletop SEMs are more than adequate for bridging the gap between optical microscopy and high-performance electron microscopes. Therefore, it is safe to say that tabletop SEMs are now on the right track towards bridging the gap between traditional electron microscopy and modern diagnostic needs. Furthermore, while molecular methods and mass spectrometry have become routine in clinical labs, they cannot replace the direct visualization of bacteria, especially when dealing with unknown pathogens or complex samples. Hence, tabletop SEM provides rapid, detailed imaging that complements these molecular techniques, offering a renewed role for EM in clinical bacteriology.

**Table 1:** Comparison of the main technical attributes of tabletop SEM, conventional SEM, and TEM relevant to their future diagnostic potential. .

Attribute	Tabletop scanning electron microscope (Tabletop SEM)	Conventional scanning electron microscope (SEM)	Transmission electron microscope (TEM)
Resolution	~10–20 nm	~0.5–15 nm	~0.1-0.5 nm
Maximum Magnification	Up to 100 thousand times	Up to 1–2 million times	Up to 50 million times
Accelerating Voltage	Limited Range 5kV-30kV	Full Range – Flexible 0.1kV-30kV	Full Range – Flexible Up to 300kV
Sample Preparation	Simple & Rapid Conductive and Non-conductive Samples	Conductive Samples	Ultrathin Sections (<150nm) Requires Embedding + Staining
Imaging Speed	Rapid (Direct Observation)	Rapid Sample and operator – dependent	Variable and labor-intensive
Depth of Information	Surface Information Elemental Composition (EDS)	Surface Information Elemental Composition (EDS)	Internal Structure Elemental Composition (EDS)
Sample Size – Thickness	Larger/thicker samples; broader field of view	Larger/thicker samples; broader field of view	Ultra-thin only; small field of view
Cost – Accessibility	Compact, affordable, user-friendly, low maintenance	Expensive, requires expertise and infrastructure	Expensive, requires expertise, infrastructure, and complex maintenance.
Diagnostic Potential	Rapid bacterial identification, Rapid AST, Direct Sample Observation.	Bacterial surface studies, bacteriophage studies, biofilm analysis.	Limited to specialized observation of internal ultrastructure and viruses with negative staining.
References	(Zmerli et al., 2023; Zmerli et al., 2024a; Zmerli et al., 2024c; High-Tech, 2025)	(Golding et al., 2016b; Hyams et al., 2020; Scientific, 2025)	(Golding et al., 2016b; Hyams et al., 2020; Scientific, 2025)

**Figure 3:**
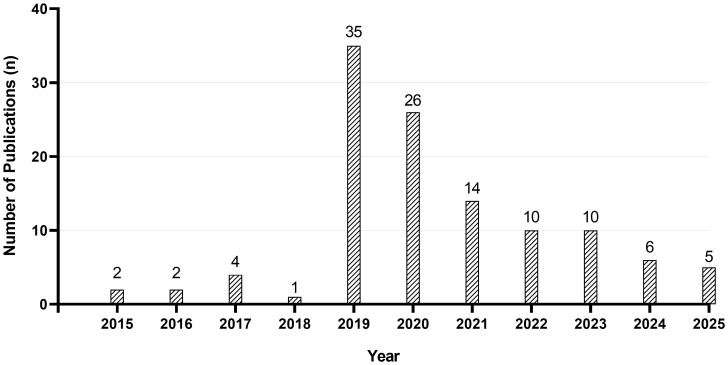
Annual number of publications that specifically report bacteriology research using tabletop scanning electron microscopes since their commercialization. (Publications included in this graph were verified by title, abstract, and full-text screening. This is a non-exhaustive list that targets the specific use of tabletop SEM for observing bacteria within the scope of bacteriology.).

In this review, we explore the major applications of tabletop scanning electron microscopy within the field of bacteriology over the last decade (2015–2025) (**Figure 3**) with a focus on the practical applications of tabletop SEM in bacteriology, highlighting its advantages and limitations and discussing how these factors may influence the future integration of this technology into diagnostic tool development. We examine key scientific publications that have shown the impact of tabletop SEM in both basic bacteriology research and in clinical bacteriology.

## 2 Basic bacteriology research applications

Studies that used tabletop SEM for basic bacteriology research included fundamental explorations of bacterial biology, including bacterial detection and visualization, biofilm formation, and bacterial interactions with environmental factors. These studies effectively demonstrated the power of tabletop SEM in capturing detailed morphological information following simple sample preparation protocols.

### 2.1 Bacterial morphology, taxonomy, and new species description

Tabletop SEM has proven its essential role in the modern study of bacterial morphology, taxonomy, and the description of new species. The largest surge in publications using SEM in the description of new bacterial species occurred in 2019, with more than 30 articles including tabletop SEM into the workflow for isolating, characterizing, and formally describing novel bacterial taxa from diverse human and environmental sources (Togo et al., 2016b; Togo et al., 2016a; Alou et al., 2017; Diop et al., 2017; Anani et al., 2019; Belkacemi et al., 2019b; Bellali et al., 2019b; Bellali et al., 2019a; Bonnet et al., 2019; Fall et al., 2019; Francis et al., 2019; Kuete et al., 2019a; Kuete et al., 2019b; Lo et al., 2019a; Lo et al., 2019b; Lo et al., 2019c; Mekhalif et al., 2019; Ndiaye et al., 2019a; Ndiaye et al., 2019b; Ndongo et al., 2019b; Ndongo et al., 2019c; Ndongo et al., 2019a; Niang et al., 2019b; Niang et al., 2019a; Takakura et al., 2019d; Takakura et al., 2019a; Takakura et al., 2019c; Takakura et al., 2019b; Tall et al., 2019; Yimagou et al., 2019b; Yimagou et al., 2019a; Yimagou et al., 2019c; Anani et al., 2020; Belkacemi et al., 2020; Benabdelkader et al., 2020b; Benabdelkader et al., 2020a; Bordigoni et al., 2020a; Bordigoni et al., 2020b; Boxberger et al., 2020b; Boxberger et al., 2020c; Boxberger et al., 2020d; Boxberger et al., 2020a; Camara et al., 2020; Dahmana et al., 2020; Gouba et al., 2020a; Gouba et al., 2020b; Konate et al., 2020; Ngom et al., 2020b; Ngom et al., 2020a; Takakura et al., 2020; Tall et al., 2020b; Tall et al., 2020a; Tall et al., 2020c; Tall et al., 2020d; Yimagou et al., 2020; Boxberger et al., 2021c; Boxberger et al., 2021a; Boxberger et al., 2021b; Khedher et al., 2021; Mbaye et al., 2021; Naud et al., 2021; Sarr et al., 2021; Li et al., 2022; Sarr et al., 2022). These studies showcased high-resolution images that reveal the surface structure, shapes, and arrangements of bacterial cells. This approach is particularly valuable in the context of culturomics (Abdallah et al., 2017; Diakite et al., 2020), where large numbers of previously uncultured or uncharacterized bacteria are isolated from the human microbiome, such as gut, skin, urine, and vaginal samples. Moreover, the evolution of the taxonogenomics approach which involves whole-genome sequencing coupled to morphological and phenotypic characterization for the description of new bacterial species was a major complementary method to culturomics, providing a comprehensive species description based on genomic data, morphological features, biochemical properties, and phylogenetic analyses (Fournier and Drancourt, 2015; Abdallah et al., 2017). SEM imaging complements molecular and phenotypic analyses by providing direct visual evidence of cell size, surface topography, and cellular organization, which are often critical for taxonomic resolution when genetic differences are subtle (Sarr et al., 2021). Moreover, SEM has facilitated the description of bacteria from unique or challenging environments, and bacteria isolated from clinical samples like urine, sputum, and dental plaque (Fall et al., 2019; Kuete et al., 2019b; Lo et al., 2019a; Niang et al., 2019b; Takakura et al., 2019b; Yimagou et al., 2019c; Benabdelkader et al., 2020a; Bordigoni et al., 2020a; Konate et al., 2020; Takakura et al., 2020).The consistent use and integration of tabletop SEM in these workflows underscores its accessibility, speed, and the minimal sample preparation required, making it an indispensable tool for taxonomists. By bridging the gap between molecular identification and classical morphology, tabletop SEM not only accelerates the discovery of new bacterial diversity but also enriches our understanding of microbial evolution and adaptation across varied ecological niches.

### 2.2 Biofilm research and bacterial interactions

In biofilm research, tabletop SEM has been used to offer high-resolution insights into biofilm architecture, bacterial distribution, and the structural changes that occur in response to various experimental conditions and environmental interventions. Several studies relied on tabletop SEM, from the development of anti-biofilm nanoparticles to the biodegradation of plastics and the investigation of bacterial joint infections.

In the context of nanoparticle-induced biofilm disruption, chitosan-propolis nanoparticles were engineered to target Enterococcus faecalis biofilms, which are common causative agents of surgical site infections. SEM imaging in these studies revealed a clear physical disruption of the biofilm matrix following nanoparticle treatment. The images showed collapsed biofilm structures and a notable reduction in bacterial aggregation, particularly at higher nanoparticle concentrations (200–300 µg/mL). This structural damage correlated with a significant decrease in bacterial viability, as confirmed by complementary viability assays (Ong et al., 2017).

Another study examined the formation and development of *Y. enterocolitica* biofilms, showing the adhesion and maturation stages of this biofilm using SEM. This allowed the discovery of a distinct form of colonies (S-form) that favors biofilm development, characterized by dense microcolonies and a thick extracellular matrix. The SEM images highlighted specific structural features, such as radial striation in S-forms and rugged edges in R-forms, which were consistent with quantitative measurements of biofilm strength (Lenchenko et al., 2019).

In prosthetic joint infection models, SEM was employed to visualize *Staphylococcus aureus* biofilms on implant surfaces. One study proved how *Staphylococcus aureus* biofilms can form protective aggregates in synovial fluid, allowing them to withstand even high doses of antibiotics like cefazolin. Structural barriers within the biofilm, such as fibrin-based aggregates, were shown to impede antibiotic penetration (Dastgheyb et al., 2015). Further studies demonstrated the utility of SEM to perform direct visualization of biofilms on extracted material, within the scope of a rat model to study *S. aureus* biofilms on prosthetic material (Irwin et al., 2023).

### 2.3 Material-bacteria interactions and device testing

Another application of tabletop SEM included the study of bacterial interaction with new material (such as coatings, membrane, or nanomaterials) that are being developed for medical and industrial use. One major aspect of these studies includes the determination of the antimicrobial properties of such materials, for which tabletop SEM was used to assess bacterial adhesion, colonization, and the effectiveness of anti-adhesive or antimicrobial modifications.

In the creation of chitosan-functionalized poly(ϵ-caprolactone) (PCL) nanofibrous membranes, tabletop SEM was used to simultaneously visualize the morphology of the electrospun fibers and to observe bacterial adherence and colonization (Mao et al., 2019). This study showed that these membranes, especially when enhanced with silver nanoparticles, significantly reduced the attachment and proliferation of both Gram-negative and Gram-positive bacteria. SEM images clearly revealed fewer bacterial cells and disrupted colonies on the surface of chitosan/PCL membranes compared to controls, proving the antibacterial effect and the relationship between fiber structure and composition in resisting bacterial colonization (Mao et al., 2019). Similarly, in the development of gelatin dressings impregnated with bioactive compounds from *Copaifera* sp., SEM was used to assess the distribution of bacteria on the surface after exposure to pathogens. These analyses helped confirm the antibacterial properties of the impregnated dressings, as fewer bacteria were observed on treated materials compared to untreated controls (Pascoal et al., 2020). Furthermore, a study of palladium nanoparticles synthesized by laser ablation in liquids for antimicrobial applications also used SEM to observe the interaction between bacteria and nanoparticle-coated surfaces. When *Staphylococcus aureus* was exposed to titanium discs coated with palladium nanoparticles, SEM images revealed clear evidence of bacterial inhibition, with fewer bacteria adhering to and surviving on the treated surfaces. This direct observation confirmed the bactericidal activity of the palladium nanoparticles and provided visual proof of their effectiveness (Fernández-Arias et al., 2022).

Phage-mediated biofilm dispersal was also investigated with SEM, by visual description confirming that quorum sensing-induced prophage activation in *Pseudomonas aeruginosa* leads to significant biofilm disruption, cell lysis, extracellular matrix degradation, and dispersal of bacterial cells. This enabled direct structural evidence for phage-mediated biofilm dispersal and the identification of potential horizontal transfer of virulence genesNdiaye et al., 2019b.

Antibacterial coating is a valuable strategy for reducing hospital-acquired infection rates while minimizing side effects and avoiding the emergence of antibiotic resistance. A study on the development of leather coatings for the prevention of diabetic foot infections adopted a SEM imaging approach for the direct observation of bacterial colonization on leathers treated with silver oxide coatings, revealing a dramatic reduction in bacterial adherence and proliferation on coated leathers compared to untreated controls. The high-resolution images allowed researchers to confirm the uniformity and integrity of the antimicrobial coating and showed that bacteria present on the coated surfaces often displayed signs of structural damage, including disrupted cell walls. This direct visualization provided compelling evidence of the coating’s bactericidal effect and underscored the value of SEM in evaluating the antibacterial performance of functionalized materials intended for medical applications (Carvalho et al., 2022).

An important review on the methodologies used for the study of infected vascular grafts highlighted the use of SEM in experimental models to visualize bacterial adhesion and biofilm development on vascular biomaterials. SEM images provided straightforward evidence of early bacterial colonization, biofilm maturation, and the impact of antimicrobial coatings, helping to elucidate infection mechanisms and the effectiveness of anti-infective strategies (Puges et al., 2023).

Other works used tabletop SEM to visualize the direct interaction between bacteria and Cu-TiO_2_-coated surfaces. The images showed that bacteria in contact with the nanocomposite coatings exhibited membrane damage and cell lysis, supporting the observed long-term antibacterial activity and demonstrating the efficacy of Cu-TiO_2_-coating at the single-cell level (Chen et al., 2024). SEM was also used to visualize biofouling on reverse osmosis membranes before and after treatment with iron nanoparticles and plasma-activated water. The technique revealed a substantial reduction in bacterial biofilms and the presence of lysed bacterial cells on treated membranes, providing visual confirmation of effective biofouling removal and improved membrane cleanliness (Egorova et al., 2024).

The use of SEM within the materials development field is vast and ever expanding (Collins et al., 1993; Romero, 2007), however the main application for SEM is restricted to observing and characterizing the surface of materials under development for topographic descriptions (Accioni et al., 2022; Fernández-Arias et al., 2022; Chadha et al., 2024), with very few studies using SEM for actual bacterial visualization and morphological description (Accioni et al., 2022; Gallingani et al., 2023). Therefore, we must emphasize that researchers must explore the use of SEM for both surface studies and for observation of bacteria and other living structures, especially with the emergence of simple sample preparation protocol suitable for such investigations (Mateiu et al., 2016; Ali et al., 2021; Czerwińska-Główka and Krukiewicz, 2021; Malyshev et al., 2024).

### 2.4 Drug development and molecule testing

Tabletop SEM has equally entered the world of drug development and molecule testing, offering direct visualization of bacterial morphology, membrane integrity, and biofilm architecture in response to novel compounds and drug delivery systems. Several studies have used this tool to correlate structural changes in bacterial cells with the efficacy and mechanisms of candidate molecules, providing both qualitative and quantitative insights that are critical for advancing therapeutic strategies.

In the investigation of streptococcal dTDP-L-rhamnose biosynthesis enzymes, SEM imaging was used to examine *Streptococcus mutans* strains with mutations in the rmlB and rmlC genes, revealing severe cell swelling and clumping, clear indicators of cell wall defects resulting from disrupted rhamnose biosynthesis (Beek et al., 2019). These morphological abnormalities provided direct evidence that these enzymes are essential for bacterial viability and confirmed them as promising targets for antibacterial drug development. Another study explored the bactericidal mechanism of butanoic acid against *Staphylococcus pseudintermedius* and *Acinetobacter baumannii* where treated bacterial cells displayed pronounced structural damage, including membrane breaches and cell lysis. These findings confirmed that butanoic acid exerts its antimicrobial effect by disrupting membrane integrity and inducing cytosolic acidification, supporting its potential as a broad-spectrum antibacterial agent (Kennedy et al., 2019).

Another study focusing on bacteriocins, specifically a novel broad-spectrum bacteriocin “Y19-2” produced by *Lactobacillus sakei*, used SEM to directly observe the bactericidal effects of Y19–2 on *Staphylococcus aureus* and *Escherichia coli*. SEM imaging revealed extensive morphological changes, including the formation of distinct pores within the bacterial cell membranes and pronounced disruption of the cell envelope integrity. This provided compelling visual evidence that Y19–2 exerts its antimicrobial activity primarily through membrane permeabilization and physical damage to the bacterial envelope, which aligns with the described mode of action for Y19–2 which involves invoking membrane damage to gain access to DNA and RNA to achieve growth inhibition (Fu et al., 2022).

Studies also reported the use of tabletop SEM in the development of drug delivery systems. One study describes its use in the development of 3D-printed microencapsulated *Lactobacillus rhamnosus* for oral administration, confirming the structural integrity and uniform distribution of microcapsules within the printed matrices. This ensured that the encapsulated probiotics remained viable and protected until reaching their target site in the gastrointestinal tract.

At the level of molecule discovery and repurposing, studies described the use of tabletop SEM for antibacterial activity detection. The antibacterial activity of coumarin-3-carboxylic acid (3-CCA) against Acidovorax citrulli was characterized using both SEM and transmission electron microscopy (TEM) (Zhu et al., 2023). SEM images showed that 3-CCA treatment led to significant membrane disruption and inhibited flagellar growth, resulting in reduced bacterial motility and impaired biofilm formation. These visual observations provided strong support for the compound’s dual-action antibacterial mechanism. Further, the design and synthesis of novel coumarin-3-carboxylic acid derivatives containing a thioether quinoline moiety were evaluated using SEM. Treated Acidovorax citrulli cells exhibited shrinkage, membrane rupture, and inhibited biofilm formation, confirming the potent antibacterial activity of these new compounds at the cellular level (Zhang et al., 2024).

Similarly, the effect of Matricaria aurea essential oils on *Pseudomonas aeruginosa* biofilms was directly visualized by SEM, which revealed a significant reduction in biofilm biomass following treatment. Sub-inhibitory concentrations of the essential oil were sufficient to disrupt the biofilm’s structural integrity, which highlights the resolving power of SEM in providing a clear observation that allows the detection of such minute details.

Lastly, the inhibitory effects of *Trichoderma asperellum* culture filtrates against *Burkholderia pseudomallei* demonstrated strong bactericidal activity, with visual confirmation of membrane damage by SEM. SEM images revealed that untreated bacteria maintained smooth, intact cell surfaces, while exposure to the filtrate caused dose-dependent morphological damage. At sub-inhibitory concentrations, bacteria appeared shrunken and displayed irregular surface textures, whereas at bactericidal concentrations, cells exhibited pronounced membrane disruption, blebbing, and deep surface lesions (Roopkhan et al., 2025).

## 3 Clinical bacteriology applications

SEM is an increasingly valuable tool in bacteriology, enabling rapid imaging of hydrated and unfixed microbes while preserving ultrastructural details and minimizing artifacts (Golding et al., 2016b). Recent technical advances, such as the use of ionic liquids and improved filtration, have enhanced SEM’s sensitivity, making it valuable for identifying bacteria—often to the genus level—and for investigating novel or emerging pathogens when the causative agent is unknown (Goldsmith and Miller, 2009). SEM also offers operational advantages over transmission electron microscopy (TEM), including faster workflows and easier sample screening, making it essential for studying bacterial surface structures, bacteriophages, and infection processes in both research and clinical diagnostics (Beniac et al., 2014). Recently, several studies performed at specialized centers with access to tabletop SEMs have demonstrated the feasibility of integrating these instruments into microbiology labs for various explorations in clinical bacteriology with immense potential for development of routine clinical diagnostics, with emphasis on automation, high-throughput protocols, and reduced sample preparation time. These studies have proposed Tabletop SEMs as a tool to replace or complement existing clinical workflows, such as Gram staining, optical microscopy, and culture-based AST. The evidence demonstrates that tabletop SEM is not only a powerful diagnostic and research tool but also a catalyst for new workflows in clinical microbiology laboratories, aligning with the global effort of finding ways to accelerate clinical microbiology diagnostics.

### 3.1 Direct-from-sample applications

Tabletop SEM was studied as a robust alternative to traditional optical microscopy and gram staining to perform direct examination of clinical samples, especially for the study of positive blood cultures. A study by Haddad, et al. systematically compared SEM to Gram staining for the identification of bacteria and yeasts directly from positive blood cultures. The findings revealed that SEM could distinguish microbial morphotypes up to the genus level in many cases, and offered a significant reduction in turnaround time, with identification possible within an hour of blood culture positivity. This rapid workflow was validated against MALDI-TOF/MS, the current reference standard, with 96.6% accuracy in correctly classifying microorganisms, representing a significant improvement over traditional Gram staining, which suffers from inter-operator variability and false staining issues. The low preparation costs and the potential for full automation of sample preparation, image acquisition, and identification further prove the potential of this new tool. The tabletop SEM approach reduced time-to-results to approximately one hour for twelve blood cultures simultaneously, while providing superior morphological detail compared to optical microscopy. The authors concluded that SEM-based identification could revolutionize urgent microbiological diagnostics and infectious disease management by enabling faster, more accurate detection and characterization of pathogens directly from clinical material (Haddad et al., 2021a).

Similarly, the ability of SEM to visualize morphotypes and structural features at high resolution was assessed for the detection of unusual or fastidious organisms that may be missed by conventional methods. For example, SEM enabled the detection and morphological characterization of spiral-shaped Treponema species in oral samples, with the entire process, including sample preparation, imaging, and analysis, completed in approximately 15 minutes. This rapid detection was critical for subsequent culture, molecular identification, and database enrichment with new spectra for MALDI-TOF MS, underscoring SEM’s role in expanding the diagnostic toolkit for rare or emerging pathogens (Belkacemi et al., 2019a).

### 3.2 Antimicrobial susceptibility testing applications

Another emerging application of tabletop SEM in clinical bacteriology is exposing its role for the development of rapid phenotypic antimicrobial susceptibility testing. Traditional AST methods are constrained by long incubation periods, often requiring 18–24 hours or more to yield actionable clinical results (Prinzi, 2024). Recent studies have demonstrated that the use of tabletop SEM can enable the detection of ultrastructural changes in bacteria exposed to antibiotics within as little as 30 to 120 minutes, enabling unprecedented real-time discrimination between susceptible and resistant strains (Haddad et al., 2021c; Bellali et al., 2022a; Zmerli et al., 2024b).

For Gram-negative bacteria, SEM was used to monitor morphological changes following exposure to imipenem, a carbapenem antibiotic often used for the treatment of MDR infections and in critical care. The method involved measuring bacterial length, width, and electron density before and after antibiotic exposure. Susceptible strains exhibited characteristic swelling, elongation, and increased electron density, while resistant strains maintained their baseline morphology. This approach was validated on 71 reference and clinical isolates and was further confirmed in a blind test of 58 clinical samples, achieving 100% correct classification of susceptibility or resistance within two hours (Haddad et al., 2021c; Hisada et al., 2023a). These results are a steppingstone towards the rapid detection of carbapenem resistance in Enterobacterales, facilitating prompt therapeutic decisions and improving patient outcomes.

Such concepts were also extended to include AST for Gram-positive cocci. A study examining vancomycin susceptibility described the use of tabletop SEM for the visualization of septation patterns and surface area changes in *Enterococcus* and *Staphylococcus* isolates after antibiotic exposure for brief time periods under 3 hours (Bellali et al., 2022a). Susceptible strains showed reduced septation and increased cell size, while resistant strains exhibited multiple septa and maintained division. These phenotypic markers were detectable within an hour of antibiotic contact, corroborating the similar findings for gram-negative bacteria. In both cases, the authors elucidated the potential development of automated AI-driven AST assays for rapid clinical implementation (Haddad et al., 2021c; Bellali et al., 2022a; Hisada et al., 2023a).

Subsequently, an innovative approach was uncovered for the determination of bacterial viability by means of a heavy-metal staining technique which uses Phosphotungstic acid (PTA) staining combined with tabletop SEM imaging for detecting live and dead bacteria in a sample. This technique enhanced the ability to visualize microscopic deformations while distinguishing bacterial cell envelope damage based on single-cell contrast changes. The staining principle relies on tungsten density increasing backscattered electron intensity, where brighter bacterial contrast indicates dead bacteria. The method was applied to a wide range of Gram-positive and Gram-negative species, demonstrating its concordance with classic viability assays including culture-based colony counts and fluorescent microscopy dye-based assays (Hisada et al., 2023a; Zmerli et al., 2023).

Researchers also integrated the use of this bacterial viability assay with previous AST findings to develop a tabletop SEM-based assay for observing morphological and quantitative viability changes in Gram-negative bacteria exposed to Colistin. 44 reference strains were tested with Colistin, stained with Phosphotungstic acid (PTA), and imaged using a tabletop SEM. The results showed that colistin-susceptible strains exhibited significant morphological alterations (inflation, fusion, and lysis), and a significant decrease in viable cells starting as early as 30 minutes after exposure. Resistant strains showed minimal changes even after 120 minutes of incubation. These findings were verified and confirmed using the gold standard broth microdilution method (Zmerli et al., 2024a).

Complimentary to performing AST, one study examined the mechanistic understanding of the bactericidal and bacteriostatic activity of Imipenem and Doxycycline, respectively, to prove the utility of SEM in predicting these effects within 2 hours of exposure to the antibiotic, as compared to the results obtained with traditional culture-based methods (Zmerli et al., 2024b). For the bactericidal antibiotic Imipenem, SEM revealed a significant reduction in bacterial density, extensive cell lysis, disrupted and deformed cell envelopes, and loss of structural integrity, which were confirmed by increased single-cell contrast following the use of PTA staining. In contrast, exposure to the bacteriostatic antibiotic Doxycycline resulted in a clear inhibition of bacterial growth, with a stable bacterial density, preserved cell morphology, and minimal ultrastructural changes, indicating growth arrest without bacterial death. These observations were concordant with traditional culture-based methods but provided results more than 16 hours earlier. Therefore, it was established that SEM can rapidly and accurately characterize antibiotic activity by integrating quantitative (bacterial density), qualitative (morphology), and viability-based criteria at the single-cell level (Hisada et al., 2023a; Zmerli et al., 2024b).

Moreover, studies coupling tabletop SEM with energy-dispersive X-ray (EDX) spectroscopy, an innovative detection technology that allows elemental composition analysis, evaluated bacterial viability and metabolic state following antibiotic exposure. This coupled methodology was used to confirm the PTA stain localization at the single-cell level, proving the “viability stain” concept that was described (Hisada et al., 2023b; Zmerli et al., 2023). Also, by quantifying elemental composition dynamics including shifts in magnesium, potassium, and sodium, and tracking Mg²^+^ dissipation in stressed bacteria this coupled approach proved to provide early predictions of bacterial viability and stress response, offering a novel complementary approach to conventional viability assays and expanding the utility of SEM for AST applications (Haddad et al., 2022).

### 3.3 Sterility testing & vaccine development

Sterility testing and vaccine development remain critical components of pharmaceutical manufacturing and clinical product safety, yet traditional methods are limited by numerous challenges including complex protocols and excessively long incubation times. While not yet standard practice, tabletop SEM has been explored as a tool for rapid sterility assessment. One study developed a rapid preparation-free method for sterility testing using tabletop SEM that facilitated the direct visualization and confirmation of *Klenkia terrae* contamination in germ-free mice units. This contamination was not detected using metagenomic and universal 16S rRNA PCR methods due to extraction and sequencing biases. This approach allowed the researchers to easily observe bacterial morphologies and structures in stool samples within minutes, supporting its utility as a complementary tool alongside gram staining for routine sterility monitoring and rapid detection of microbial contaminants in laboratory animal facilities. The authors highlight that combining SEM with traditional and molecular methods can enhance the reliability and speed of sterility testing, particularly in settings where fastidious or difficult-to-detect bacteria may be present (Andreani et al., 2020).

Other studies have utilized tabletop SEM to assess the morphology and surface characteristics of bacterial and vaccine microparticles in the scope of vaccine development. Specifically, SEM enabled detailed examination of the structural integrity and uniformity of inactivated *Neisseria gonorrhoeae* microparticles (Gala et al., 2018), confirming that the formalin-fixed whole-cell bacteria retained their intact, native morphology and that the resulting microparticles displayed irregular, rough surfaces with varied shapes and sizes (mostly 1–5 µm in diameter) (Gala et al., 2018). This use of tabletop SEM provided essential quality control data, ensuring that the physical properties of the vaccine candidates met the necessary standards for further immunological evaluation. Likewise, other researchers attempted to identify surface morphology and structural features of *Staphylococcus aureus*, visualizing bacterial cell surfaces and confirming the presence and accessibility of candidate antigens identified through proteomic and immunological analyses. This imaging supported the selection of vaccine targets by providing direct evidence of their surface localization and potential exposure to the host immune system during infection (Reijer et al., 2017).

### 3.4 Investigation of infective endocarditis and urinary tract pathologies

Studies elucidating the pathogenesis of complex bacterial infections such as infective endocarditis (IE) have also used tabletop SEM for the ultrastructural analysis of infected heart valve tissue. This revealed unique patterns of cellular organization, biofilm formation, and elemental composition associated with different bacterial pathogens, even when histological analysis was negative. Researchers examined five IE vegetations and a control valve, revealing distinct differences in bacterial abundance and organization: *Enterococcus faecalis* was present in low amounts, while *Staphylococcus aureus*, *Streptococcus oralis*, *Streptococcus agalactiae*, and *Streptococcus gallolyticus* were found in larger numbers, with notable observations such as dividing *S. oralis* cells showing double bodies and septa. Notably, SEM combined with EDX analysis demonstrated that vegetations associated with viridans streptococci contained calcium-phosphate deposits resembling hydroxyapatite, suggesting that these bacteria may promote species-specific patterns of calcification in heart tissue, potentially influencing the physical consistency and embolic risk of the vegetations (Hannachi et al., 2020). Furthermore, this combined approach enables rapid stain-free detection and chemical characterization of IE, offering a valuable tool for improved diagnostic strategies beyond classic pathology. These findings also have implications for therapy, as they can inform the selection of antimicrobial strategies. The integration of SEM with histopathological and immunohistochemical techniques further enhances the characterization of infected tissues, allowing for the localization of bacterial antigens, the identification of immune cell infiltrates, and the correlation of structural changes with clinical outcomes. Such multimodal approaches are advancing the understanding of host-pathogen interactions in IE and other deep-seated infections.

Furthermore, some studies focused on performing detailed urinalysis and urinary stone analysis using SEM coupled to EDX. Yacouba et al. demonstrated that this combined technique is highly effective for the direct detection and identification of both microorganisms and crystals in urine samples. The study included over 200 urine samples that were imaged using SEM, producing high-resolution images that allowed for the morphological classification of bacteria (including gram-negative bacilli, cocci, and yeasts), epithelial cells, leukocytes, and erythrocytes, while EDX offered precise elemental identification of urinary crystals. Calcium oxalate was the most frequently detected crystal, followed by struvite and others, with the elemental composition (e.g., C, O, Ca for oxalate; Mg, P, O, N for struvite) confirming the crystal types. From a wider perspective, Costa-Bauzá et al. presented a specialized protocol utilizing stereoscopic microscopy, SEM, and infrared spectroscopy for the morpho-compositional study of kidney stones, demonstrating how SEM allows for rapid, highly reliable identification of stone components, including those present in very small proportions or within complex, non-homogeneous samples. This approach provided a detailed visualization of both external and internal stone structures, including those adhering to renal tubules. Another investigation into papillary renal calculi characterized five distinct types of papillary stones, each with unique structural and compositional features, using a combination of SEM imaging and elemental analysis. The study highlighted that oxidative stress in papillary tissues can promote heterogeneous nucleation, leading to the crystallization of calcium phosphate and calcium oxalate. SEM paired with EDX enabled the precise differentiation of stone types—such as calcium oxalate monohydrate and dihydrate, uric acid, and mixed stones with urate or apatite deposits. This detailed characterization is crucial for understanding the pathogenesis of stone formation and the influence of urine composition and tissue environment. Therefore, these works have shown how SEM coupled to EDX could identify and characterize crystals and microorganisms that are often missed or misidentified by routine light microscopy, providing a more comprehensive urinalysis, especially valuable in cases of complex or ambiguous findings.

### 3.5 Microbiome explorations

The applications of tabletop SEM extend beyond individual bacterial analysis to studies of more complex microbial ecosystems. Tabletop SEM was used for the study of novel nano-bacterial structures characteristic of Candidatus Saccharibacteria (TM7) across a variety of human sample types, including oral, fecal, breast milk, vaginal, urine, and infectious samples (Naud et al., 2023). SEM imaging enabled the identification of CPR-like morphologies in all sample types except cardiac valves, providing morphological confirmation of these bacteria complementing molecular detection methods. This direct visualization was critical for verifying the presence and distribution of Candidatus Saccharibacteria in both commensal and clinical settings, supporting their role as widespread members of the human microbiome (Naud et al., 2023). Other research pioneered the use of this instrument for performing an accurate investigation of gut microbiota composition in a dysbiosis-related disease model. This study established and optimized a rapid and reproducible protocol for preparing stool samples for tabletop SEM analysis, enabling high-resolution imaging of gut microbiota components. SEM was used to capture over 40,000 micrographs from 40 stool samples, allowing for the manual identification, classification, and morphometric analysis of bacteria (bacilli, cocci), yeasts, and other objects within this ecosystem. The approach facilitated the direct comparison of microbiota diversity and morphological integrity between controls and samples from patients with *Clostridioides difficile* infection, revealing disease-associated shifts such as reduced bacilli and increased yeasts (Boukili et al., 2025).

## 4 Discussion and perspectives

**Figure 4:**
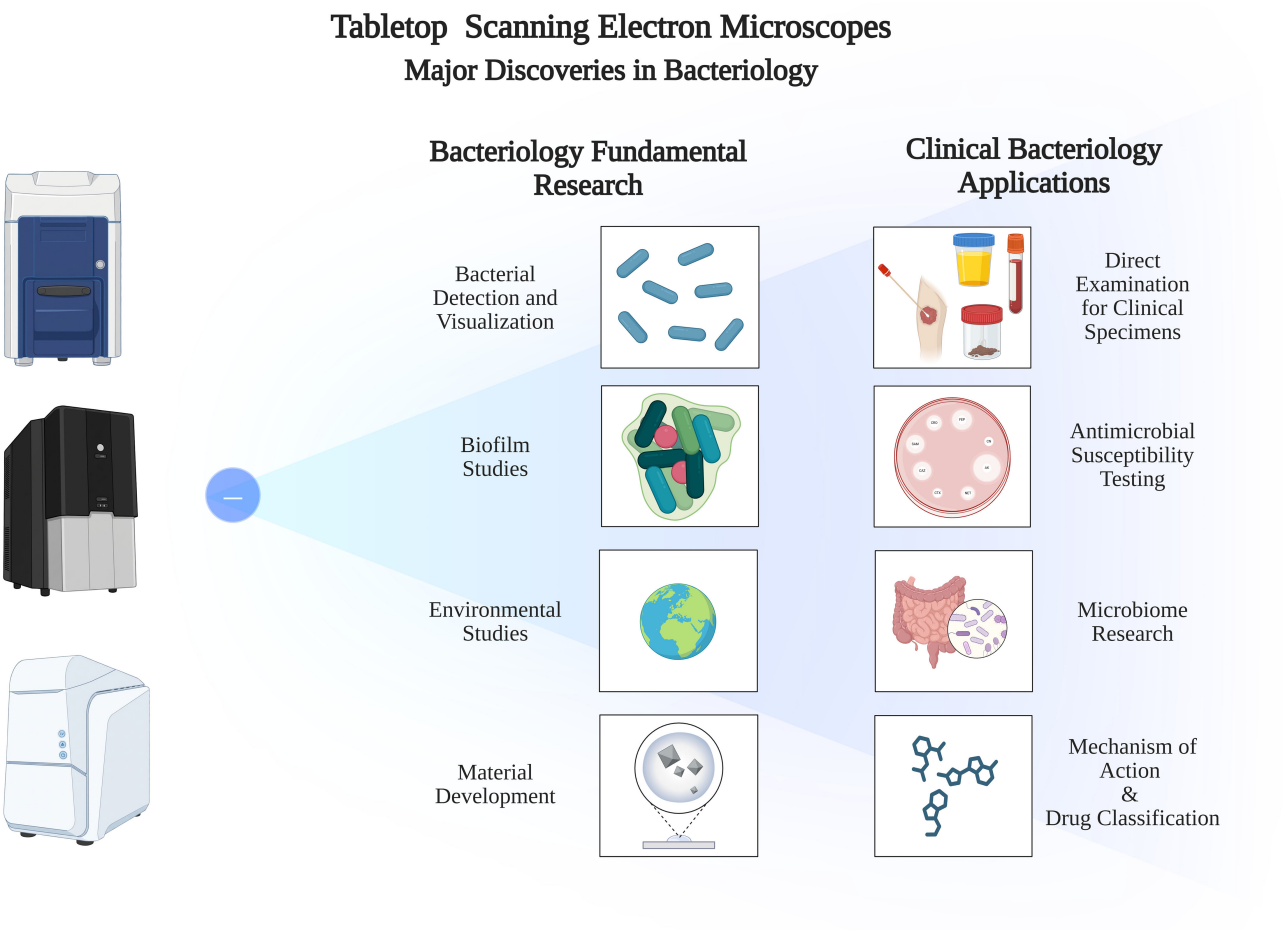
Major discoveries in bacteriology using tabletop scanning electron microscopes.

In the past decade, there has been considerable evidence supporting the utility of tabletop SEMs in the vast field of bacteriology, both at the research and clinical levels, as illustrated in **Figure 4**. The combination of the instrument’s accessibility, efficiency, and high-resolution imaging positions it as an indispensable tool for the future. Starting at the level of fundamental research, we explored how tabletop SEM is emerging as a versatile and multifaceted tool capable of generating diverse data across a range of disciplines, including bacteriology, materials science, and environmental microbiology. A major promising application lies in the study of biofilms, where tabletop SEM can enhance rapid access to details related to bacterial distribution, biofilm architecture, formation mechanisms, maturation processes, and high-resolution visualization of extracellular structures that help maintain biofilm integrity. This knowledge extends to impact translational research, where insights derived from SEM observations can be applied to clinical challenges including the management of device-associated infections, the design of antimicrobial surfaces and material, and the exploration of mechanisms underlying antimicrobial resistance. These fundamental applications are also valuable for environmental and ecological explorations, where tabletop SEM integration can significantly advance our understanding of the role of bacterial communities in environmental settings, by uncovering bacterial survival strategies and environmental adaptation. This promising potential is certain as traditional SEM technology has already made its impact in a range of industrial fields, already achieving robust results at the research level (Harvey and Edwards, 2022). For example, in the food production industry, SEM was used for investigating foreign body contamination in food products, identifying contaminants with sizes ranging from 1–1000 um (James, 2005). Likewise, quality control SEM applications have also explored quality of ingredients and packaging alike (Pretorius and Buys, 2020; Luiz L da et al., 2021). SEM technologies have also been useful in agricultural applications, especially for plant disease diagnosis through pathogen visualization, including ultrastructural evaluations of cellular arrangements and surface structures allowing a comparative analysis with molecular data (Khiyami et al., 2014; Cubero et al., 2024). Various other environmental applications including water quality assessment were also extensively explored by SEM for biofilm detection and monitoring water treatment demonstrating the benefits of direct biofilm observation (Alhede et al., 2012; Ogura, 2014; Trinh et al., 2025). Therefore, it is important that further research targets the creation of rapid and simplified protocols for performing these analyses using tabletop SEM, which is expected to reduce the time to results without compromising image quality. Likewise, SEM applications in the pharmaceutical industry have emerged over the years (Klang et al., 2013; Harvey and Edwards, 2022), with major impact on drug formulation characterization, stability assessment, quality control, and drug cytotoxicity explorations (Depalo et al., 2019). In addition, recent advances in AI-driven in silico drug discovery could potentially integrate SEM for accelerating the *in-vitro* testing of candidate drug effects and physicochemical properties at the single-cell level (Iwata, 2023; Hornick et al., 2024). Therefore, there is serious potential for developing SEM applications that leverage the power and rapidity of tabletop SEM for accelerating many of these processes.

While more efforts are needed to develop basic bacteriological research applications, it is equally important to recognize the significant advancements already achieved in clinical bacteriology, notably at the proof-of-concept level. From rapid diagnostic testing to complex microbiome analysis, tabletop SEM has proven its value as a dynamic tool for deepening our understanding of bacteriology, especially at the level of bacterial detection, visualization, viability analysis, and bacteria-antibiotic interactions. These applications hold significant potential to enhance clinical outcomes, particularly as they are further developed into practical diagnostic tools that simplify existing workflows. Their scope is expected to continue expanding as researchers design new protocols and explore hypotheses that were previously unattainable due to the limitations of traditional methods (Eldin et al., 2019).

Tabletop SEM has allowed researchers to overcome the major challenges related to bacterial detection in clinical samples, including blood cultures (Haddad et al., 2021b). This has also been extended to more complex challenges including the development of SEM-based antimicrobial susceptibility testing protocols enabling the observation of antimicrobial effects at significantly earlier time-points, as compared to conventional AST methods that rely on culture or other indirect detection methods (Haddad et al., 2021d; Bellali et al., 2022b; Hisada et al., 2023b; Zmerli et al., 2024a). The innovative coupling of tabletop SEM with powerful complementary methods such as EDX for elemental composition analysis has also introduced a new dimension to bacterial exploration. This approach must be further emphasized as such chemical-level insights could hold significant clinical relevance for elucidating host-microbe interactions and insights on homeostasis. Therefore, future research in clinical bacteriology must build upon these promising foundations to develop robust clinical diagnostic tools capable of providing rapid data-rich answers to clinicians.

However, this target is undeniably linked to the necessity of integrating process automation and artificial intelligence (AI) models for expediting access to results. This creation of more “smart” platforms will enable a shift from manual workflows toward automated AI-enabled systems. These systems will offer automated sample handling, standardized acquisition protocols and high-throughput automated imaging, generating large high quality datasets (Kalinin et al., . 2023; Morone and D’Antuono, 2025). However, a major bottleneck persists at the level of image analysis. Large datasets should be analyzed in real-time by dedicated machine learning models (Kalinin et al., . 2023). In an era increasingly shaped by advancements in AI and machine learning (ML), and given their successful integration into image analysis across various microscopy techniques, there is a serious need to apply these approaches to SEM (Alsulimani et al., 2024). SEM-specific algorithms could be trained to perform automated bacterial detection, morphological classification, and recognition of subtle structural patterns associated with antimicrobial activity or resistance; effectively transforming each microscope into an intelligent decision-support tool within the diagnostic pathway (Zmerli et al., 2024b). This exploration is particularly warranted, as SEM offers inherently high-resolution imaging and facilitates unambiguous identification of distinct structural features; capabilities that often surpass those of lower-resolution modalities such as optical microscopy (Haddad et al., 2021b). Such integration was a recurring perspective cited in most of the analyzed studies. Therefore, future works focused on the development of AI/ML-based analysis in this context holds significant promise for accelerating and enhancing the rapid analysis of SEM data.

In parallel with advances in data analysis, substantial progress can be achieved by building fully automated sample preparation workflows. Many current protocols already rely on sequential and highly reproducible steps that are inherently compatible with robotic handling and microfluidic integration, making them strong candidates for high-throughput platforms. Transitioning from manual to automated preparation would enable simultaneous processing of multiple samples, reduce hands-on time, improve inter-operator reproducibility, and bring SEM-based assays closer to the turnaround times expected in routine microbiology (Boukili et al., 2025). Integrating these automated tools into existing laboratory workflows, including direct interfacing with blood culture systems and sample tracking infrastructure, will be a critical milestone toward future clinical adoption.

It remains critical to note that tabletop SEMs present certain technical limitations that are challenges to be overcome in case they are to replace traditional techniques, particularly optical microscopy. This is highlighted by the need for further development to improve high-throughput image acquisition, by reducing the scanning time needed to obtain the high-resolution images. Achieving truly high-throughput imaging will require further optimization of beam control, detectors, and scanning strategies to shorten acquisition times without compromising resolution, as well as smarter acquisition schemes that dynamically adapt magnification and field selection based on real-time image analysis. This is essential for the clinical applications as it represents a key determinant enabling SEM to compete with other imaging techniques.

Another priority is the development of robust methodologies for imaging hydrated and partially hydrated specimens, including bacteria in physiological fluids and complex biofilms. This is vital for capturing bacteria in their native environments and for conducting in-depth studies of their metabolic dynamics. As these hardware and protocol innovations mature, they should be accompanied by systematic efforts to evaluate tabletop SEM performance across diverse bacterial species, life-cycle stages, and ecological niches. Likewise, increased efforts are needed to develop protocols that enable researchers to use tabletop SEM to more effectively describe variability among bacterial populations and genera, at the level of life cycle, pathogenicity, and broader bacteriological phenomena. Such approaches will help identify the remaining limitations of tabletop SEM in the bacteriology field and stimulate the exchange of knowledge between bacteriologists and the SEM development industry.

In conclusion, ongoing technological advancements are central to the advancement of bacteriology. For tabletop SEM, we believe that the continued development of advanced research applications will uncover previously unseen microbial environments. This progress will foster the emergence of specialized rapid diagnostic assays adopting a syndromic approach, suitable for point-of-care applications in resource-limited settings. In these contexts, combining cost-effective, rapid, and simple assays will be essential for diagnosing and treating acute diseases such as sepsis and meningitis, thereby improving patient outcomes and survival. These discoveries and diagnostic breakthroughs will reaffirm and secure the return of SEM to the evolving landscape of microbiology.
